# Challenges and lessons learnt from the ENJOY project: recommendations for future collaborative research implementation framework with local governments for improving the environment to promote physical activity for older people

**DOI:** 10.1186/s12889-021-11224-7

**Published:** 2021-06-22

**Authors:** Pazit Levinger, Jeremy Dunn, Maya Panisset, Briony Dow, Frances Batchelor, Stuart J. H. Biddle, Gustavo Duque, Keith D. Hill

**Affiliations:** 1 National Ageing Research Institute, Royal Melbourne Hospital, PO Box 2127, Melbourne, Victoria 3050 Australia; 2 Institute for Health and Sport, Victoria University, Melbourne, Australia; 3 Rehabilitation, Ageing and Independent Living (RAIL) Research Centre, Monash University, Melbourne, Australia; 4 Centre for Health Policy, University of Melbourne, Melbourne, Australia; 5 School of Nursing and Midwifery, Deakin University, Waurn Ponds, Australia; 6 Department of Physiotherapy, The University of Melbourne, Melbourne, Australia; 7 Centre for Health Research, University of Southern Queensland, Springfield, Queensland Australia; 8 Australian Institute for Musculoskeletal Science (AIMSS), The University of Melbourne and Western Health, Melbourne, Australia

**Keywords:** Physical activity, Older people, Implementation, Seniors Exercise Park, Outdoor

## Abstract

**Background:**

The physical environment has been shown to have a positive effect on the promotion of physical activity of older people. Outdoor environments that incorporate specialised exercise equipment suitable for older people are uniquely placed to promote physical activity and social connectedness amongst older people. The ENJOY project included the installation of specialised outdoor exercise equipment (the Seniors Exercise Park) and the delivery of a physical and social activity program for older people as part of a prospective pre-post research design. The installation of the specialised equipment in public sites and an aged care facility was also aimed at increasing usage of the equipment by older people from the wider community and to increase physical and social activities.

**Method:**

A conceptual framework for implementation and several engagement methods were utilised to guide the research and to support the participating partners throughout the project. This paper is a reflective narrative describing the collaborative process and approach utilised to engage local governments and community, and reports the challenges and the lessons learnt to inform future strategies for implementation.

**Results:**

The conceptual framework for the implementation process that guided the conduct and delivery of the ENJOY project included the core elements of the Interactive Systems Framework and the ecologic framework. These models incorporate elements of research-to-practice and community-centred implementation to accommodate the unique perspectives of a range of stakeholders.

**Conclusion:**

Partner characteristics such as local governments’ structure and policy as well as community factors can impact on implementation. Partnership with local governments with effective communication, strategic planning and community and seniors engagement approaches are recommended for successful implementation. The lessons learnt can further assist public health research design around changes to the built environment to positively impact on older people’s physical activity levels.

**Trial registration:**

Trial registration number ACTRN12618001727235. Date of registration 19th October 2018, https://www.anzctr.org.au/Trial/Registration/TrialReview.aspx?id=375979

## Introduction

Physical inactivity is associated with many chronic diseases, morbidity and mortality [1] with more than a quarter (1.4 billion) of the world’s adult population not getting enough physical activity to maintain their health [2]. In most countries fewer than half of older adults are active enough to achieve the health benefits associated with physical activity [3]. In Australia, only 25% of people aged 65 years and over meet the recommended physical activity guidelines [4].

The physical environment has been shown to have a positive effect on the promotion of physical activity in older people [5]. The outdoor environment, including walkability, accessible green spaces, exercise equipment and amenities are important factors to facilitate engagement in physical activity [6]. These features are of particular importance to the maintenance of activity levels in older age as older people are likely to be impacted by their local environment and outdoor neighbourhood conditions [7, 8]. Outdoor exercise equipment has become quite common in recent years as important environmental infrastructure to provide opportunities for physical activity and social connectedness in public areas [9–11]. However, these are rarely designed to meet the needs of older people. The design of outdoor space and associated physical activity equipment needs to be considered carefully to better suit the ageing population [6]. For example the following aspects are important to consider in outdoor exercise equipment to suit the older demographic: the inclusion of handrails for support (if required), the platform height (to be similar to a standard step/stairs height or lower), and the addition of equipment stations that target balance (unstable/uneven surfaces), mobility and daily functioning movements (e.g., stairs, sit to stand, range of movement). Inclusive and accessible outdoor environments that incorporate age-specific specialised exercise equipment have great potential to promote physical activity and social connectedness amongst this demographic.

**Fig. 1:**
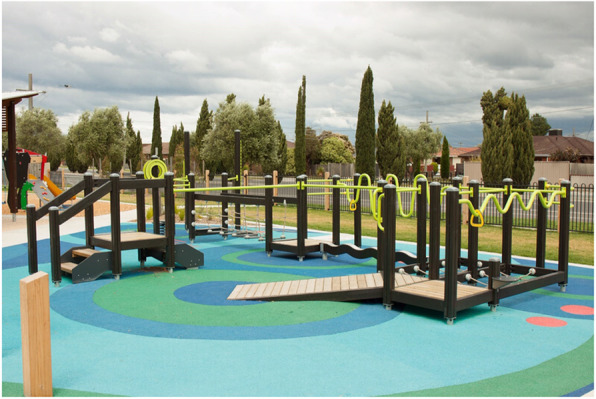
The Seniors Exercise Park at Thomastown, Melbourne. The picture was taken by the lead investigator

In 2018 we commenced an active ageing partnership project (The ENJOY project: Exercise interveNtion outdoor proJect in the cOmmunitY) that was designed to fulfil the need for an age-friendly outdoor space for older people to be physically active in the community [12–14]. Active ageing has been defined by the World Health Organisation as “ the process of optimizing opportunities for health, participation and security in order to enhance quality of life as people age” [15]. The ENJOY project was based on our previous research that demonstrated the effectiveness of the Seniors Exercise Park program on improving physical function and social health in older people [16, 17]. The ENJOY project involved two Victorian local Councils (Wyndham and Whittlesea City Councils) and another partner agency (Old Colonists’ Association of Victoria, a residential aged care and seniors living provider). The project included the installation of specialised outdoor exercise equipment (the Seniors Exercise Park, Fig. 1) together with the delivery of physical and social activity program for inactive older people (age 60 years and over) as part of a prospective pre-post research design [12, 13]. In contrast to some other existing outdoor exercise equipment in community parks that mostly focus on strength and or fitness exercise (gym-like machines), the Seniors Exercise Park incorporates multiple exercise stations that include balance, strength, function, flexibility and dexterity exercises [18]. Project timelines included 6–12 months preparation and discussion with local governments followed by 18–22 months for the delivery of the research project at each participating site. The overarching aims of the ENJOY project were: (1) to promote the innovative outdoor exercise equipment as a physical activity opportunity for older people and (2) to enable independent uptake and delivery of physical activity programs utilising the Seniors Exercise Park by local city Councils and seniors’ organizations. Importantly, the installation of the specialised equipment in public sites and within an aged care and independent living facility also aimed to facilitate increased usage by older people from the wider community. Outcomes of the ENJOY project demonstrated that participation in the physical activity program using the Seniors Exercise Park resulted in sustained engagement in physical activity, improved physical function, wellbeing, quality of life and physical activity level for older people [13, 14]. A conceptual framework for implementation and several engagement methods were utilised to guide the research and to support the participating partners throughout the project.

The aim of this paper is to provide a reflective narrative describing the collaborative process and approach utilised to engage local governments and community, and to report the challenges and lessons learnt to inform future strategies for implementation. It is believed that the lessons learnt can further assist public health programs and research on the built environment to positively impact on older people’s physical activity levels. We describe the conceptual framework for implementation that incorporates several engagement methods, key challenges, and associated strategies and recommendations.

### Conceptual framework

**Fig. 2:**
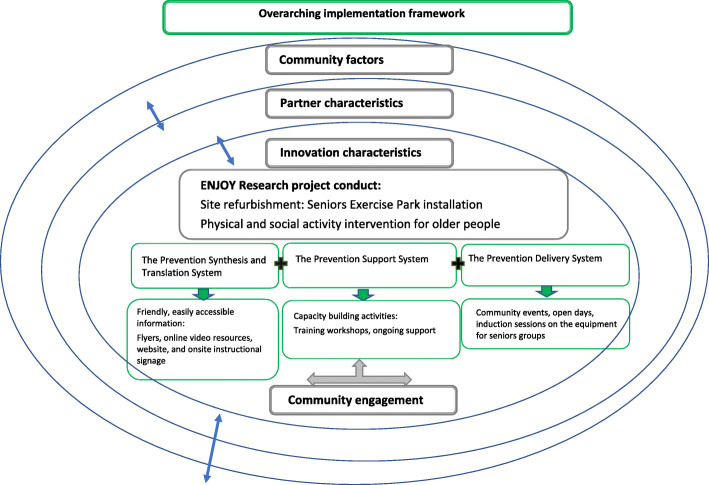
The overarching implementation framework is presented using the ISF and within the larger context of partner characteristics and community factors (ecological approach). The implementation of the innovation (The Seniors Exercise Park) is guided by the Prevention Delivery System and its organizational capacity, the Prevention Support System and the Prevention Synthesis & Translation System. Interactions between all components occurred throughout the research trial. Adapted from the American Journal of Community Psychology, Volume 41(3–4), Durlak J.A and DuPre E.P. Implementation Matters: A Review of Research on the Influence of Implementation on Program Outcomes and the Factors Affecting Implementation, 327–350, Copyright (2008), with permission from John Wiley and Sons

Transferring effective programs (effective interventions) into real world settings and maintaining them longer term can be a complicated and lengthy process that requires dealing effectively with the successive, complex phases of program diffusion (from dissemination, adaptation, implementation and sustainability) [19]. Identification of the factors that affect the implementation process is important to enable a framework to be established to guide a successful research implementation process and hence sustainability [19]. The conceptual framework for the implementation process that guided the conduct and delivery of the ENJOY project included the core elements of the Interactive Systems Framework (ISF) and the ecologic framework [19, 20]. These models incorporate elements of research-to-practice and community-centred implementation to accommodate the unique perspectives of a range of stakeholders (Fig. 2). The principal elements considered essential for successful implementation of the ENJOY were partnerships with local governments and stakeholder engagement [21].

#### The ISF consists of three systems (set of activities) [20]

The first system, Prevention Synthesis and Translation System, involves distilling information about the innovations (e.g., programs, policies, processes, and principles) and preparing it for implementation by end users. Within the ENJOY project, this involved distilling information about the Seniors Exercise Park and how to use it in a clear, friendly and easily accessible manner for older people participating in a research project and those from the wider community. This included flyers with exercise instructions, online video resources of the exercises, online information on the organisations’ website, and onsite instructional signage.

The Prevention Support System is conceptualized as supporting the work of those who will put the innovations into practice, carrying out as two primary support functions: innovation-specific support (innovation specific capacity-building) and general support (general capacity-building). Within the ENJOY project, this was provided through training for allied health professionals such as Accredited Exercise Physiologists/Physiotherapists/qualified Exercise Instructors (specific-capacity building) and ongoing support and training for staff within the Council (general capacity-building to build skills, and motivation of the organization). We delivered 6 separate training workshops that included theoretical and practical sessions incorporating, safe usage guidelines, risk management, exercise prescription, and delivery. Supporting hard copy resources were also provided (exercise sheets and illustrations, exercise cards). The workshops were delivered by the research team members (Accredited Exercise Physiologist and Physiotherapist) in 2019 as follows: three workshops in Whittlesea City Council, Thomastown (2nd April, 30th April, 14th October); two in Wyndham City Council, Hoppers Crossing, (7th May, 25th Sep) and one in Leith Park residential aged care (14th May) with a total of 130 attendees.

The Prevention Delivery System is defined as the individuals, organizations, and communities that carry out prevention delivery activities which have varying levels of existing capacity (both ability and motivation) to implement prevention. Within the ENJOY project, this consisted of site refurbishment (equipment installation and associated capital work) and the activities initiated by the local government and/or local leisure/health care providers related to usage of the Seniors Exercise Park by residents via programs or other modes of delivery. Both Councils had funds available for site refurbishment (capital works) but the cost of the exercise equipment was covered by the research fund. This approach provides a strong incentive for partnership and collaboration. Moreover, the availability of resources such as staff and or (other) funding played a major role in the extent of activities by the partners. For example, the refurbishment of the park by Wyndham City Council was supported by funding from the Victorian State Government. This enabled the upgrade of the adjunct community centre and other supporting promotional activities surrounding the Seniors Exercise Park.

#### Ecological framework approach

The three critical components of the ISF are embedded within a larger context of provider characteristics and community factors. Interactions between all components are expected and required for successful implementation (Fig. 2), consistent with an ecological framework [22]. Due to the nature of the project and the partnership with local governments, the organizational structures, characteristics and capacity are important to consider as they are likely to be responsible for guiding the implementation of a new program [19]. The Seniors Exercise Park was installed by the participating partners, and they hold the overall responsibility for its maintenance and usage, and hence are the key driver to promote usage and facilitate the delivery of physical activity programs.

The following sections describe important considerations around the provider/partner characteristics and community factors (ecological framework) that were identified as directly impacting on implementation within the context of the ENJOY project. We therefore detailed the situation, the approach utilised to overcome potential hurdles and consequently the lessons learnt. A Summary is provided in Table 1.

**Table 1:** Summary of Study specific challenges underpinning the implementation framework and associated recommendations

Study specific challenges underpinning the implementation framework	Lessons learnt and future recommendations
**Partner/provider characteristics**	
*Local government structure and communication* Departments within local governments sometimes work in silos, with lack of reciprocal communication in the process of the park revitalisation plan	Understand the structure within the Council and identify the relevant teams/departments in the initial planning phase (Infrastructure and Active Ageing) Early identification of which department will be responsible for site management
*Partnership agreement*	A formal agreement is recommended to seal commitment and expectations of both parties
*Policy for shade cover for playground* Often lack of funding and policy can hinder installation of safe shade cover	Educate and advocate to Council staff to adopt/update shade policy and ensure urban planners incorporate shade into outdoor site design
*Timing flexibility and budget constraints* Lack of alignment between research conduct timing and site construction work	Advanced planning should take place while taking into account the lengthy internal process required by local governments Adapt a flexible approach to the study design for a realistic and practical execution
**Community factors**	
*Priority area for safe use of specialised outdoor equipment* Equipment might be used by other age groups which can create clashes and safety risks for older people	Priority signage at the site and clear communication are needed to create public awareness for safe usage of all age groups
*Community engagement and seniors involvement*	Encourage engagement of senior ambassadors and involvement of end users throughout the research project
*Promotion and communication strategies specific for older people* Online platforms might not be accessible by older people	Identification of strategies that are relevant for older people for better community reach are recommended

## Partner/provider characteristics

### Local government structure and communication

There are various departments within local governments that have had different roles in the context of the Seniors Exercise Park initiative. Upgrade or development of an outdoor recreation area is usually the responsibility of the Community Infrastructure team/department (e.g., Strategic planner, Landscape and Open Space, Public Space/Urban Design of Open Space). This department will be responsible for the actual site design, location within a park, construction and associated infrastructure requirements, and choice and allocation of outdoor equipment. On the other hand, the Age and Disability, Positive/Active Ageing team, Sport and recreation, or equivalent is responsible for the delivery of services for older people including physical activity programs.

The demand on capital and recurrent expenditure by local governments can be problematic especially around ongoing management and maintenance. While it is often relatively easy to find one-off grants or funds for capital expenditure, it is more difficult to find the recurrent spend needed to maintain equipment and the environment around it to a high standard in subsequent years. However, it should be noted that the long term commitment by Councils for this ongoing expenditure for Seniors Exercise Parks is no different to what they currently commit to and fund for children’s playgrounds and other Council infrastructure. The approached utilised in the ENJOY project was to partner with local governments that have already secured funds for site refurbishment (capital works) and have factored in the maintenance costs required longer term. This approach ensured that the local governments have had the appropriate planning and associated budget in place.

#### Challenges and lessons learnt

These departments sometimes work in silos, with little communication or consultation with each other in the process of park revitalisation plans. This created some difficulties in communication as some aspects of the ENJOY project required coordination between different departments at various stages of the project. Discussion with all relevant departments should ideally take place at the initial planning phase so the design of a site/park upgrade/construction will take into consideration input from the Positive/Healthy Ageing team to suit the older demographic. It is also important to understand and determine which department is responsible for the overall management of the exercise equipment utilisation beyond the completion of park refurbishment.

### Partnership agreement

Conducting a research project in the community requires partnership with local governments and community groups for longer term engagement and sustainability. Links between industry and local governments, and academic/research institutions are important to facilitate research translation [23].

#### Challenges and lessons learnt

For the ENJOY project, each partner organisation had a positive ageing strategy (or equivalent) in place which aligned with both organisational priorities and hence the overall aim of the project. A positive ageing strategy is an approach that incorporates positive attitude around ageing [24] that guides many local governments to promote the health and wellbeing of residents and provides opportunities for older residents to maintain social connectedness and remain active in their local communities. A memorandum of understanding was signed between the lead research organisation and each partner to formally seal the expectations and requirements for the conduct of the project. This overarching agreement provided an anchor point for commitment to overcome any unexpected changes within Council structure (e.g., staff change).

### Policy around shade cover for playground

Exercising outdoors offers many health benefits, but can equally present barriers due to weather elements [25]. Purpose-built shade may increase use of parks while providing effective and sustainable ways to reduce UV exposure of community populations [26].

#### Challenges and lessons learnt

In the ENJOY project the two public sites had no cover at the beginning of the project, while the third site (aged care site) had cover installed prior to commencement of recruitment at that site. Initial discussions took place to flag the need for a shade cover to protect older people from the weather elements. However, Councils’ policy often favours natural shade (e.g. trees) as the most common form of shade for most playgrounds. While natural shade is the preferred option, mature trees do not always already exist at sites. The discussion around the addition of a shade cover encountered concerns around the short life span, susceptibility to vandalism and the maintenance cost. It was therefore important to educate Council staff and provide research evidence as well as ongoing feedback from the participants to support the need for a shade cover. Community members also independently approached the Council directly via a petition to advocate the need for cover for safe usage. Midway through the project, a sail shade was installed by Wyndham City Council followed by a late installation (after completion of the project) by Whittlesea City Council. Adopting suitable shade policy is needed to ensure that urban planners include shade cover into outdoor venues design and allocate the financial resources to support this.

### Timing flexibility and budget constraints

Commonly, research projects will have a defined timeline and an allocated budget for the conduct and completion of the project.

#### Challenges and lessons learnt

Working with local governments and stakeholders requires considerable flexibility as there are internal processes that need to take place and are outside the control of the research team. The process for budget approval for infrastructure work within local governments may take up to 12–24 months prior to construction work actually commencing. This timeline may not work well with research funding and reporting requirements. These challenges are frequently experienced in natural experimental designs where the actual intervention is a site/park refurbishment or any form of infrastructure upgrade [27, 28]. In the ENJOY project installation of the Seniors Exercise Park was planned to take place in three sites but at different periods. Hence, the research commencement did not necessarily align with construction work completion. We therefore staggered recruitment commencement one site at a time to accommodate completion of the construction work. This flexible staggered approach has enabled us to capitalise on the unavoidable variation in installation periods and worked well in the overall execution of the project. It is also important to note that contact with the participating Councils for the ENJOY project was made with long lead times of almost a year, which enabled planning in advance as well as securing external funds to conduct the project.

## Community factors

### Competing interests and priority area for safe use by older people

Designing an outdoor space that includes adjacent intergenerational zones for both outdoor equipment for older people and playground for children is an important consideration to allow all generations to be physically active and to foster intergenerational interaction. The Seniors Exercise Park is a multigenerational play equipment that can be utilised by all ages.

#### Challenges and lessons learnt

In both public sites, a children’s playground was adjacent to the Seniors Exercise Park. In Whittlesea City Council, the park was also located close to a kindergarten and towards the end of kindergarten sessions, children and parents attended the park. This created some clashes where children wanted to play on the equipment while exercise classes were running and posed a safety risk for older people who participated in the classes. While public space can be accessed and used by all residents, consideration of safety of all age groups is important. There are no local laws relating to priority use of public facilities by different groups, however local governments’ position is that the group for which the equipment is designed has priority use. To overcome potential clashes, Whittlesea City Council communicated to the kindergarten staff and families, explaining that during designated times precedence should be given to older people. Priority signage at the site and clear communication to create public awareness are recommended for safe usage of all age groups.

### Community engagement and seniors’ involvement

Engagement with the community and end-user members (older people) is important for sustainability as well as in the design of scalable public health interventions [29]. Community engagement has been recognised as a pathway to building trust, encourage participation, and promote the uptake of findings [30].

#### Challenges and lessons learnt

To foster engagement we informally invited participants who showed interest in being involved in advocacy (ENJOY champions (ambassadors)) to join community events, other research activities (promotional resources e.g., video production) and mentorship to other seniors participants/residents. This involved three participants at Thomastown (Whittlesea City Council), five in Hoppers Crossing (Wyndham City Council), and two in Leith Park (aged care site). The ENJOY champions took part in open day events, Seniors’ Week, Community Health Expo events and more. The involvement of ENJOY champions as ambassadors greatly improved communication and engagement of older people from the community to increase participation in Council’s programs, activities and events. A positive ageing reference group and/or age friendly ambassadors are quite common in local governments and served to provide feedback to Council on its policies, plans and services. In particular, they can provide invaluable advice to Council in relation to communication, engagement and consultation with older people, and assist in promoting the benefits of positive and active ageing. Therefore, engagement of senior ambassadors and involvement of end users throughout the research project is recommended.

### Ongoing promotion to create awareness -strategies specific for older people

Communication and promotion of the Seniors Exercise Park to older people was done using several media platforms. The approach utilised varied between the partners and mainly included online platform such as information on websites, hard copy flyers placed at the community centre and other Councils centres (e.g., libraries).

#### Challenges and lessons learnt

The effectiveness of using online information to create awareness of the specialised equipment and hence to reach older people to use it might have been limited. Access to online information and resources might be limited by lack of knowledge or skills in using internet and smartphone as well as lack of internet access by older people, which is relatively common in older people [31]. Flyers placed at central points, visits to seniors events and seniors groups seem to work better. Similarly, promotion via ENJOY champions (ambassadors) and engagement and involvement of older people with various research activities is also likely to result in greater reach. Consequently, careful consideration needs to be taken when designing health promotion campaigns for older people so the communication and marketing strategies are suitable and likely to be taken up by the end users.

### Generalisability of the approach

This paper discusses specific recommendations associated with the conduct of the ENJOY project for older people, many aspects can also be utilised to guide successful collaborative research work with local governments and other stakeholder organisations that are focussed on other populations and activities. These include effective communication, formal written agreements, clear mutual objectives, flexible timelines, and clear plans around longer term management and maintenance commitment.

### Corona virus diseases of 2019 (COVID19) implications

The COVID19 pandemic has created various challenges with many restrictions put in place by governments around public outdoor access, travel, physical distancing and more. While the activities detailed above were not impacted by COVID19, COVID19 did impact on subsequent community use of the Seniors Exercise Park during lockdowns. In Australia, in 2020 the Victorian State Government enforced restrictions around access to public parks, outdoor exercise equipment and play space. This prevented community members from using and accessing the Seniors Exercise Parks with many programs being unavailable to access (e.g., put on hold or ceased until further notice) during the lockdown. In late 2020, restrictions were gradually eased and access to parks and outdoor exercise equipment were permitted. In early 2021 most restrictions have been lifted and programs have been resumed. Although engagement in indoor physical activity (indoor gyms/recreational facilities) has been difficult during the pandemic, outdoor activities have offered a safer option for physical activity with low risk of virus transmission [32]. Importantly, the pandemic has raised the importance of outdoor public spaces and parks to people's wellbeing and health and hence access to parks and outdoors should be encouraged [33]. Several recommendations have been proposed for safe access and usage of outdoor space during the pandemic with appropriate safeguards in place [34]. These include, for example, park access monitoring, appropriate messaging, evaluation and policy updates, maintenance of physical distancing while using the equipment, usage of hand sanitisers and regular equipment cleaning routine.

## Conclusion

The ENJOY project included a unique set up of specialised outdoor exercise equipment installation and the delivery of associated physical and social activity programs for older people. As such, it offered a great opportunity for community engagement and the promotion of physical activity for older people from the wider community. Key factors were identified to impact on implementation, which involved the provider/partner characteristics such us local government structure and internal processes as well as community factors. Partnership with local governments with effective communication, strategic planning and community and seniors engagement approaches are recommended for successful implementation.

## Data Availability

Not Applicable.

## Abbreviations

ENJOY: Exercise interveNtion outdoor proJect in the cOmmunitY
ISF: Interactive Systems Framework (ISF)
COVID19: Corona virus diseases of 2019
